# Nebulizer Care and Inhalation Technique in Children with Cystic Fibrosis

**DOI:** 10.3390/children7100153

**Published:** 2020-09-27

**Authors:** Argyri Petrocheilou, Athanasios G. Kaditis, Evgenia Troupi, Ioanna Loukou

**Affiliations:** 1 Cystic Fibrosis Department, Agia Sofia Children’s Hospital, Thivon and Papadiamantopoulou, 115 27 Athens, Greece; tzenitzeni2017@gmail.com (E.T.); ioannaloukou@gmail.com (I.L.); 2 Division of Pediatric Pulmonology, First Department of Pediatrics, National and Kapodistrian University of Athens School of Medicine and Agia Sofia Children’s Hospital, Thivon and Papadiamantopoulou, 115 27 Athens, Greece; kaditia@hotmail.com

**Keywords:** nebulizers, cystic fibrosis, inhalation technique, disinfection

## Abstract

Nebulizers are used by the great majority of cystic fibrosis patients for delivery of cornerstone treatments. Inhalation technique and adequate disinfection and maintenance are important for optimizing medication delivery. In this study, inhalation technique and nebulizer disinfection/maintenance were assessed in cystic fibrosis patients by direct observation in clinic and completion of a scoring sheet. A total of 108 patients were recruited. The maximum inhalation technique score was attained by 30.5% and adequate inhalation technique score by 74.08% of patients. The inhalation technique score was best with the vibrating mesh nebulizer (*p* = 0.038), while patient age and number of nebulized medications did not affect ITS significantly (*p* > 0.05). Nebulizer disinfection/maintenance score was excellent in only 31.48%. Most families kept the nebulizer clean and used appropriate disinfection method, but only half of them replaced the nebulizer and nebulizer cup at the recommended time intervals. Nebulizer disinfection/maintenance score was positively affected by a number of nebulized medications and negatively by years of equipment use (*p* = 0.009 and *p* = 0.001, respectively). Even though inhalation technique and disinfection/maintenance practices were found to be adequate in a large proportion of cases, there is still a need for regular review and education. The type of nebulizer was associated with improved inhalation technique, but more data are required before making specific recommendations.

## 1. Introduction

Life expectancy and quality of life in cystic fibrosis (CF) patients has greatly improved in recent years as a result of aggressive treatment [1,2]. Many of the prescribed medications are delivered through inhalation and oftentimes through a nebulizer. Nebulization is time consuming, and nebulizers need to be cleaned and disinfected adding to the treatment burden [3]. Furthermore, inhalation technique is important for optimal efficacy of nebulized treatments [4,5]. It has been shown that adherence with nebulizer use and maintenance is challenging for CF patients, while inadequate disinfection can lead to bacterial and fungal contamination of the equipment [6,7,8,9].

There is often confusion on what is the best way to clean and disinfect a nebulizer, an issue that adds extra burden to patients and their families [3,10,11]. It has been reported that manufacturers’ instructions are not always consistent with guidelines for CF patients and that patients receive conflicting information from different sources [6,12,13,14]. This evidence underlines the importance of healthcare providers reviewing instructions with CF patients and their families.

Current clinical practice at our institution is that patients and their families be instructed on cleaning and disinfecting the nebulizer whenever a new device is purchased. The first dose of any nebulized medication is administered in clinic, and the appropriate technique is reviewed. Inhalation technique and nebulizer cleaning/maintenance are assessed at regular intervals. However, limited published information is available regarding the factors that affect inhalation technique and equipment maintenance. It was hypothesized that older children with better FEV_1_ percent predicted would have better inhalation technique and that nebulizer cleaning/maintenance scores would be higher if fewer inhaled medications were used. Hence, the aims of the present study were: (i) to assess patient inhalation technique; (ii) to evaluate appropriateness of nebulizer disinfection method and maintenance; and (iii) to identify factors affecting inhalation technique and nebulizer disinfection/maintenance.

## 2. Patients and Methods

### 2.1. Patients, Study Design, and Data Collection

Cystic fibrosis patients followed at the Cystic Fibrosis Department of Agia Sofia Children’s Hospital were recruited for this prospective study. The study was approved by the Agia Sofia Scientific Council on 24 May 2017, Ref Number: 3685/14-02-2017. Written informed consent was obtained from the children’s parents or patients if older than 18 years. There were no exclusion criteria. Families were contacted by phone and were instructed to bring their home nebulizer for assessment at a clinic visit or during a hospitalization. Information collected was age, nebulizer type (vibrating mesh nebulizer vs. jet nebulizer), number of nebulized medications, and FEV_1_ percent predicted during the visit. The Quanjer 2012 Global Lung Initiative Equations were used for FEV_1_ percent calculation [15].

A scoring sheet was developed to assess nebulizer disinfection methods, nebulizer maintenance, and patient inhalation technique (Appendix A). The scoring sheet comprised of three parts: (i) the general information part; (ii) the disinfection/maintenance part; and (iii) the patient inhalation technique part. In the general part, information on the type of nebulizer, year of purchase, and number of nebulized medications was obtained. In the disinfection/maintenance part, information on cleanliness/condition of the nebulizer parts, applied disinfection method, and frequency of replacement of nebulizer parts was recorded. In the inhalation technique part, patient inhalation technique and body posture during nebulization were assessed. Inhalation technique was defined as good for older children if there was good seal of the lips around the mouthpiece and the breaths were slow and deep. For younger children, technique was considered good if the mask fitted well over the mouth and nose and if the infant/young child was not crying excessively during the nebulization. If one of the above needed to be corrected, technique was considered moderate, if all technique aspects needed correction it was considered fair. A score of 3 was assigned to good inhalation technique, a score of 2 to moderate, and 1 to fair. Body posture was considered good if the child was sitting upright and the neck was not bent or overextended for older children, while for infants, it was considered good if the infant was sitting on the parent’s lap without fussing excessively. Similarly to technique, if one element of the posture assessment needed correction, then body posture was considered moderate, and if all elements needed corrections, it was considered fair. A score of 3 was assigned to good body posture, a score of 2 to moderate, and 1 to fair. Therefore, the maximum score for patient inhalation was 6 if both inhalation and posture was good, each getting a score of 3.

The same health care provider (ET) completed all assessments and assignment of scores. Questions to patients or their families were asked in an open-ended format. Inhalation technique and disinfection/maintenance scores (primary outcome measures) were calculated by adding individual scores for each of the questions (questions 4, 5, 6, 7 for disinfection/maintenance score). The maximum score for disinfection/maintenance score was 9, while a score of 6 or greater was defined as adequate.

Explanatory variables that were selected for analysis included: patient age, years of nebulizer use, number of nebulized medications, nebulizer type (vibrating mesh vs. jet nebulizer), and FEV_1_ percent predicted (for children older than 6 years). It was thought that older children and children that have been using a nebulizer for a longer time would have higher technique scores. It was also hypothesized that children with higher FEV_1_ percent predicted would have higher scores. Regarding maintenance score, higher number of medications increases treatment burden and might negatively affect maintenance scores, as the nebulizer would be used more frequently. It is unknown if nebulizer type affects inhalation technique or maintenance, however faster nebulization might improve technique, as it decreases treatment time.

### 2.2. Statistical Analysis

Three regression analysis models were tested: (i) patient inhalation technique as primary outcome and patient age, number of nebulized medications, and nebulizer type as explanatory variables; (ii) patient inhalation technique as primary outcome and patient age, number of nebulized medications, nebulizer type, and FEV_1_ percent predicted as explanatory variables in children aged >6 years; (iii) nebulizer disinfection/maintenance score as outcome measure and years of nebulizer use, number of nebulized medications, and type of nebulizer (vibrating mesh nebulizer vs. jet nebulizer) as explanatory variables.

## 3. Results

### 3.1. Patient Characteristics

A total of 108 patients were studied, with a median age of 10 years (range 2–21 years), 37% of whom were male. Ninety-one patients were older than 6 years and provided reliable spirometry results; median FEV_1_ percent predicted was 99% (range 36–147%). Thirty-six (33.33%) participants used a vibrating mesh nebulizer and the remaining 72 (66.66%) subjects used a jet nebulizer. Twenty-six (24.07%) patients were receiving one nebulized medication, 43 (39.81%) two medications, 23 (21.30%) three medications, and 16 (14.81%) four medications.

### 3.2. Patient Inhalation Technique and Nebulizer Disinfection/Maintenance Scores

The inhalation technique score was maximal in about a third of cases (31.48%). Approximately one third of patients (30.56%) attained the maximum score for nebulizer disinfection/maintenance score and approximately three quarters (74.08%) had an adequate score (6 and above). Results are summarized in Table 1.

**Figure 1:**
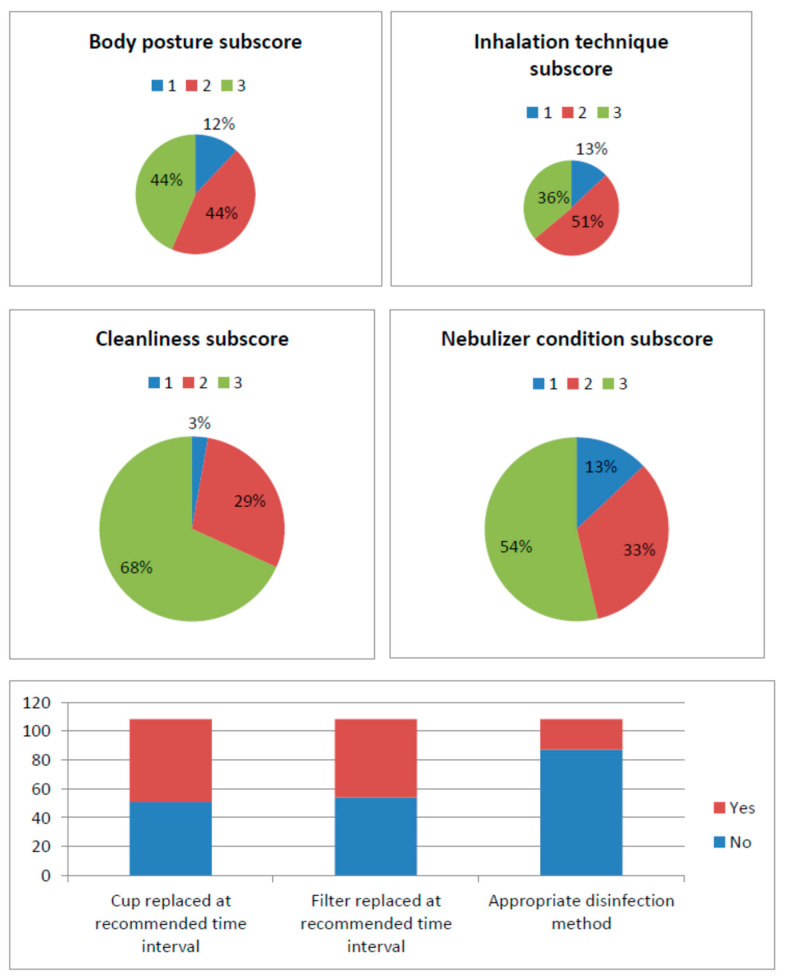
Patient inhalation technique and nebulizer disinfection/maintenance subscores.

When subscores were considered, many patients had excellent inhalation technique and posture scores, but the majority still required guidance for further improvement (Figure 1). Most families kept the nebulizer clean and used an appropriate disinfection method, but only half of them replaced the nebulizer and the nebulizer cup at the recommended time interval. The nebulizer was in perfect condition in only half of patients (Figure 1).

### 3.3. Factors Affecting the Patient Inhalation Technique Score and the Nebulizer Disinfection/Maintenance Score

Nebulizer type (vibrating mesh compared to jet nebulizer) positively affected the inhalation technique score, while patient age, number of nebulized medications, and FEV_1_ percent predicted were not significantly associated with the score (Table 2). Patients and families were more likely to have a better disinfected/maintained nebulizer if the nebulizer was newer and if more nebulized medications were administered, while the nebulizer type did not seem to have a significant role (Table 3).

## 4. Discussion

In the current study, inhalation technique and nebulizer disinfection and maintenance status were assessed in patients with cystic fibrosis by direct observation in clinic and completion of a scoring sheet. Excellent inhalation technique and nebulizer disinfection/maintenance scores were attained by only one-third of patients/families. Inhalation technique was best with a vibrating mesh nebulizer, while only half of patients/families replaced the nebulizer and nebulizer cup at recommended time intervals. Thus, nebulizer condition was negatively affected by the years of equipment use.

Inspiratory flow rate and body posture influence the surface area of lung deposition [5]. It is recommended that patients should maintain the upright sitting position when they receive nebulized medications for taking advantage of optimal lung mechanics and maximizing medication deposition [16]. Direct patient observation at home using videotaping has demonstrated that inhalation technique is frequently suboptimal [17]. This finding has been confirmed in the present study. The great majority of our patients have required further guidance to perfect their inhalation technique.

A vibrating mesh nebulizer was related to higher inhalation technique scores relative to the jet nebulizer. While type of nebulizer has been shown to affect treatment time, little is known about how the nebulizer type could affect inhalation technique in patients with cystic fibrosis [18,19]. More specifically, a mesh nebulizer reduces the duration of nebulization [18,20,21]. Although there is no specific explanation for the recognized association between technique and nebulizer type, reduced nebulization time might be a critical factor.

Interestingly, the number of nebulized medications and patient age are not associated with the inhalation technique score, indicating that adequate technique is feasible in children with cystic fibrosis regardless of their age and their treatment burden. In the subset of patients with available spirometry results, FEV_1_ percent predicted was also not associated with technique score, but this finding could be secondary to the high proportion of subjects with normal lung function in our patient cohort (median FEV_1_ percent predicted 99%).

Nebulizer cleaning and disinfection is an important aspect of care in patients with cystic fibrosis. There have been studies indicating that appropriate disinfection is related to reduced nebulizer contamination with pathogens [9,22]. Available evidence reveals that disinfection of the nebulizer once daily is adequate [23]. There are published guidelines on appropriate cleaning and disinfection techniques that are specific to patients with cystic fibrosis and that are not always consistent with the manufacturer’s instructions [4,12]. There are several approved cleaning and disinfection techniques in the guidelines; steam and hot water disinfection techniques are commonly used. There is evidence that steam and hot water disinfection are both associated with decreased nebulizer output, therefore it is important to check the nebulizer regularly to assess nebulizer function [24].

Moreover, replacement of the nebulizer cup at the recommended intervals is of paramount importance as particle formation and medication delivery change with equipment use and especially when using vibrating mesh nebulizers [20]. In the present study, the frequency of nebulizer parts replacement and disinfection methods were assessed. It was found that replacement of nebulizer parts was not performed at the recommended time intervals in half of the cases. This finding could be the result of limited family financial resources. As a result, number of years of nebulizer use was associated with worse maintenance scores.

Most families disinfected the nebulizer using one of the methods recommended in published guidelines [4,12]. In the majority of cases, a baby bottle sterilizer has been utilized as steam disinfection is an easy and effective way to disinfect nebulizer parts [10]. It should be noted that a few families (5.55%) have been using vinegar for disinfection, a previously recommended method. Vinegar is an effective disinfectant for *P. aeruginosa* but not for other important pathogens like *S. aureus* [12]. It is possible that families have received this instruction from the retailer, as it was mentioned in the manufacturer’s instructions. In response to this finding, the retail company has been contacted and been advised about this important change of practice regarding nebulizer disinfection. The instructions given by the retailer now reflect this change in practice.

Interestingly using more nebulized medications was associated with a better maintenance score. There is no clear explanation for this study finding. Higher number of nebulized treatments is often associated with higher disease severity and many of the nebulized medications are antibiotics for *P. aeruginosa* infection. Hence, we speculate that patients using more nebulized treatments might better recognize the importance of being meticulous with disinfection compared to patients with less severe disease or without *P. aeruginosa* infection.

A limitation of the present study is that family attitudes towards the importance of disinfection have not been assessed. In a recent study, a gap between recognition of the importance of disinfection and the actual consistency in following the recommended disinfection procedures was identified [21]. In addition, we have not adjusted our results for families’ socioeconomic status or education level. In a recent study on the relationship between family attitudes and nebulizer cleaning and disinfection practices, no significant difference has been shown, and thus, family attitude parameters might not have affected our results [21]. Finally, this study included patients with CF and their families attending a single CF center in Greece. Hence, it is unknown if these findings apply to other counties.

## 5. Conclusions

Even though inhalation technique and disinfection practices were found to be adequate in a large proportion of cases, there is still a need for regular review and education of patients and their families. Type of nebulizer was associated with improved inhalation technique, but more data are necessary before making a specific recommendation. Number of nebulized medications did not affect inhalation technique or disinfection/maintenance scores, underscoring that family engagement and adherence to treatment might be more important than the actual treatment burden.

## Figures and Tables

**Table 1:** Technique and maintenance total scores.

Patient Inhalation Technique Total Score, N (%)	Patients, N (%)
2	7 (6.48)
3	11 (10.19)
4	42 (38.89)
5	14 (12.96)
6	34 (31.48)
**Nebulizer disinfection/maintenance total score, N (%)**	
2	2 (1.85)
3	3 (2.78)
4	8 (7.4)
5	15 (13.89)
6	11 (10.19)
7	19 (17.59)
8	17 (15.74)
9	33 (30.56)

**Table 2:** Associations between age, number of nebulized medications, nebulizer type, FEV_1_ percent predicted (for group aged >6 years) with patient inhalation technique score.

	Regression Model 1: Inhalation Technique (Outcome Measure) *r* = 0.271; *p* = 0 .046		Regression Model 2: Inhalation Technique in Children >6 Years Old (Outcome Measure) *r* = 0.296; *p* *=* 0.092	
	Beta Standardized Coefficients	*p* Value	Beta Standardized Coefficients	*p* Value
Patient age	0.106	0.319	0.136	0.230
Number of nebulized medications	−0.005	0.963	0.068	0.530
Nebulizer type 1 = vibrating mesh nebulizer 0 = jet nebulizer	0.214	0.038	0.232	0.034
FEV_1_ percent predicted			0.100	0.380

**Table 3:** Associations of nebulizer disinfection/maintenance score with years of use, number of nebulized medications, and type of nebulizer.

	Regression Model: Disinfection/Maintenance Score (Outcome Measure) *r* = 0.434; *p* < 0.001	
	Beta Standardized Coefficients	*p* Value
Years of nebulizer use	−0.295	0.001
Number of nebulized medications	0.235	0.009
Nebulizer type 1 = vibrating mesh nebulizer 0 = jet nebulizer	0.166	0.065

**Figure A1:**
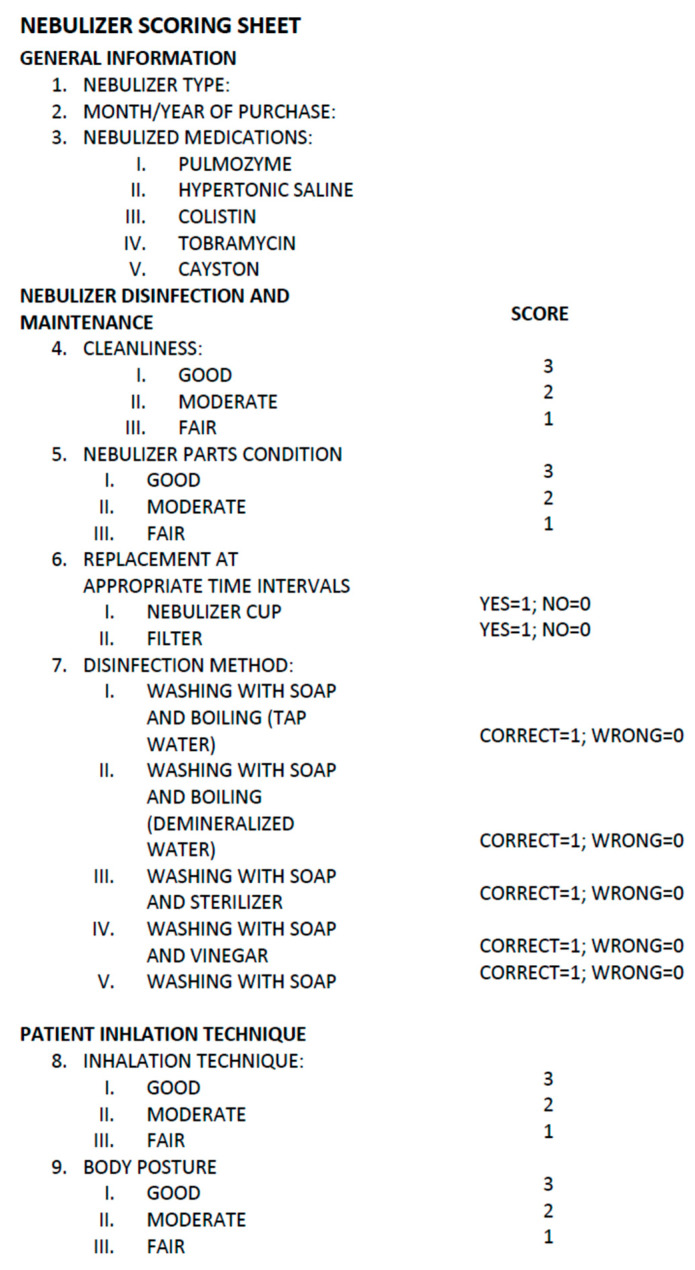
Scoring sheet Caption.
